# Public rental housing and its association with mortality – a retrospective, cohort study

**DOI:** 10.1186/s12889-018-5583-6

**Published:** 2018-05-29

**Authors:** Jun Jie Benjamin Seng, Yu Heng Kwan, Hendra Goh, Julian Thumboo, Lian Leng Low

**Affiliations:** 1 Duke-NUS Medical School, 8 College Road, Singapore, 169857 Singapore; 2 Program in Health Services and Systems Research, Duke-NUS Medical School, 8 College Road, Singapore, 169857 Singapore; 3 Faculty of Science, National University of Singapore, Singapore, Singapore; 4 Health Services Research Centre, Singapore Health Services, Singapore, Singapore; 5 Department of Rheumatology and Immunology, Singapore General Hospital, Singapore, Singapore; 6 SingHealth Regional Health System, Singapore Health Services, Singapore, Singapore; 7 Department of Family Medicine and Continuing Care, Singapore General Hospital, Outram Road, Singapore, 169608 Singapore; 8 SingHealth Duke-NUS Family Medicine Academic Clinical Program, Singapore, Singapore

**Keywords:** Public rental housing, Social determinant of health, Low socioeconomic status, Mortality

## Abstract

**Background:**

Socioeconomic status (SES) is a well-established determinant of health status and home ownership is a commonly used composite indicator of SES. Patients in low-income households often stay in public rental housing. The association between public rental housing and mortality has not been examined in Singapore.

**Methods:**

A retrospective, cohort study was conducted involving all patients who utilized the healthcare facilities under SingHealth Regional Health (SHRS) Services in Year 2012. Each patient was followed up for 5 years. Patients who were non-citizens or residing in a non-SHRS area were excluded from the study.

**Results:**

A total of 147,004 patients were included in the study, of which 7252 (4.9%) patients died during the study period. The mean age of patients was 50.2 ± 17.2 years old and 7.1% (*n* = 10,400) of patients stayed in public rental housing. Patients who passed away had higher utilization of healthcare resources in the past 1 year and a higher proportion stayed in public rental housing (*p* < 0.001). They also had higher rates of co-morbidities such as hypertension, hyperlipidaemia and diabetes. (*p* < 0.001) After adjustment for demographic and clinical covariates, residence in public rental housing was associated with increased risk of all-cause mortality (Adjusted hazard ratio: 1.568, 95% CI: 1.469–1.673).

**Conclusion:**

Public rental housing was an independent risk factor for all-cause mortality. More studies should be conducted to understand health-seeking behavior and needs of public rental housing patients, to aid policymakers in formulating better plans for improving their health outcomes.

## Background

Socioeconomic status (SES) is a well-recognized determinant of health status. Low SES influences one’s health, rate of morbidity and mortality [1]. SES influence health via the interaction between the individual’s socioeconomic characteristics as well as their area’s socioeconomic condition [2, 3]. A multitude of measures are available for assessment of SES such as home ownership, income level, educational status and occupation [4]. Some of these information are not routinely collected during healthcare encounters or comprehensively at the population level.

Public housing is a widely used composite SES measure and various studies have shown a positive correlation between public housing and poor overall health status [5–7]. Underprivileged housing condition had been associated with poorer health such as a higher prevalence of injuries, infectious diseases and chronic medical conditions [6]. For example, in the HOPE VI panel study, residents staying in public housing were found to have a two-fold risk of developing chronic medical conditions such as hypertension and hyperlipidemia [8]. Likewise, the Fragile Families and Child Wellbeing Study found that public housing residency is linked with obesity and poorer health statuses of mothers [9]. Poorer health outcomes are also contributed by overcrowding, inadequate sanitation and ventilation that result in communicable diseases. Importantly, residence in underprivileged housing is also a marker of lower SES that underpins potential social instability, and lack of access to basic healthcare [8].

Globally, home ownership has been shown to be inversely associated with mortality [10]. In Europe, this protective effect has also been shown to persist into old age [11]. The inverse relationship between home ownership and mortality has also been observed in populations such as children and African-Americans [12, 13] as well as among patients with atrial fibrillation, diabetes and stroke [14–16]. A study conducted in Finland showed that residence in rented housing has been associated with higher mortality, despite adjustment for household income, occupation and education level [17]. Home ownership may hence represent material living standards and economical wealth that is inadequately captured by conventional socio-economic indicators.

In Singapore, the majority (82%) of its population attain home ownership by purchasing public housing sold on a 99-years lease agreement [18]. Public housing locally can be broadly stratified into one to five bedrooms flats, studio apartments and executive condominiums. In 2010, the average monthly household income for citizens was SGD$7214 [19]. For households with the lowest income bracket of ≤SGD$1500 per month, public rental housing is made available by the government for rental at highly subsidized rates and this accounts for 6% of the public housing stock [20].

There has not been any study which has examined the association between public rental housing and mortality in Singapore. Locally, public rental housing residents present as a unique population with high healthcare utilization [21]. Thus, by utilizing public rental housing as an indicator of low SES, we aimed to assess the association between public rental housing and mortality risk.

## Methods

A total of six regional health systems were created by the Ministry of Health Singapore for integration of care geographically across Singapore in 2011. Among the 6 clusters, Singhealth Regional Health System (SRHS) is the largest cluster, responsible for the provision of healthcare in South-Central Singapore and also providing care for patients from other areas of Singapore. It is supported by primary care facilities such as polyclinics as well as the largest tertiary hospital in Singapore, Singapore General Hospital, which oversees over 88,000 inpatient admissions each year.

We performed a retrospective, cohort study involving patients who were under the care of SRHS and residents in the SRHS coverage area of South Central Singapore in Year 2012. Patients aged 21 years old and above were included if they were hospitalized or utilized the services of any SRHS related healthcare facilities. Each subject was followed up for 5 years.

Patients who were non-citizens were excluded as they were unlikely to be under long-term medical care from SRHS. In addition, patients who resided in non-SRHS residential areas were excluded as they would likely be under the care of a different regional health system. In Singapore, residents are eligible to rent 1 to 2 room public housing apartments at government subsidized rates if their gross household income do not exceed SGD$1500 per month. Approval from SingHealth Centralised Institutional review board (CIRB) (Reference number: 2016/2294) was obtained prior to initiation of the study.

Information pertaining to patient’s socio-demographic and clinical characteristics was drawn from electronic medical records. Socio-demographic information extracted included patient’s age, gender, ethnicity, as well as the number of patients staying in public rental housing. Codes from International Classification of Diseases (ICD) [22] were used to extract information pertaining to major co-morbidities in the Charlson and Elixhauser comorbidity index [23] such as diabetes mellitus, hypertension and renal disease etc. A total of 26 major comorbidities were extracted for this study. The healthcare utilization of each patient in the past one year was also captured as this information enabled identification of patients who were frequent users of the healthcare system [24, 25]. This data included each patient’s number of public primary care clinic visits, emergency department visits, specialist clinic visits and hospital admission. The primary endpoint in this study was all-cause mortality.

### Statistical analyses

All statistical analyses were performed with SPSS version 23 (SPSS Inc., Chicago, IL, USA). Student’s t-test and Chi-square test were utilized to examine differences between the socio-demographic and clinical characteristics of patients who were alive or died at the end of the study period, where appropriate. To assess the association between mortality with public rental housing, multivariate Cox regression analyses was performed, adjusting for age, gender, ethnicity, past one-year utilization and 26 major comorbidities. Survival probabilities from all-cause mortality were stratified by residence in public rental housing and analysed using the Kaplan-Meier curve. The results were then compared using two-sample log-rank test. A two-tailed *p*-value of < 0.05 was considered statistically significant.

## Results

**Fig. 1:**
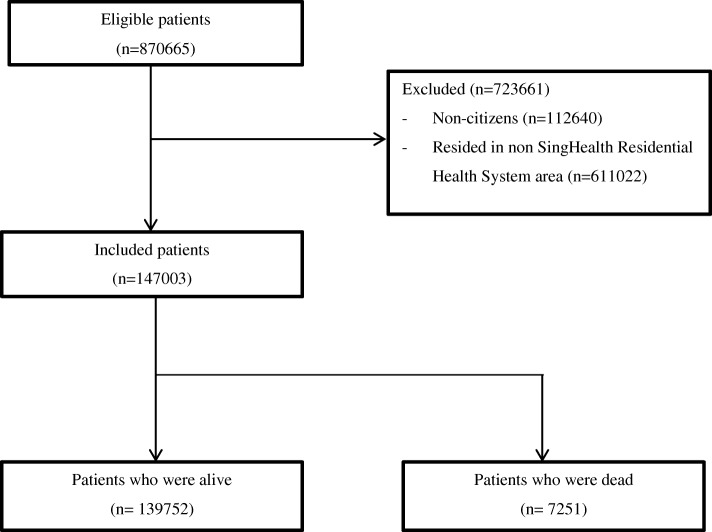
Flowchart for inclusion of patients during study period from January 2012 to December 2016

Figure 1 shows the flowchart for inclusion of a patient in this study. Of the initial 870,665 patients, 112,640 and 611,022 patients were excluded as they were non-citizens and did not reside in SHRS areas respectively. A total of 147,003 patients were included in the study, of which 7251 patients died during the study period. Each patient was followed up for a mean duration of 2.78 ± 1.55 years.

Table 1 shows the baseline socio-demographic and clinical characteristics of patients included in the study. The majority of patients were female (57.8%) and of Chinese ethnicity (78.5%), with a mean age of 50.2 ± 17.2 years. Overall, 7.1% (*n* = 10,400) of patients stayed in public rental housing. Compared to patients who were alive, patients who passed away during the study period were older, had higher utilization of healthcare resources in the past 1 year and a higher proportion stayed in public rental housing (*p* < 0.001). The rates of all 26 co-morbidities examined in the study were higher in patients who died compared to patients who were alive (*p* < 0.001).

**Table 1:** Baseline characteristics of patients (*n* = 147,003)

Characteristics	All Patients (*n* = 147,004)	Patients who died (*n* = 7251)	Patients who were alive (*n* = 139,752)	*P*-value^#^
Patient demographics				
Age, Mean (SD)	50.2 (17.2)	63.9 (16.2)	50.1 (17.2)	< 0.001
Gender, Male (%)	62,171 (42.3%)	4030 (54.8%)	58,141 (41.6%)	< 0.001
Ethnicity				< 0.001
Chinese (%)	115,456 (78.5%)	6212 (85.7%)	109,244 (78.2%)	
Indian (%)	11,263 (7.7%)	439 (6.1%)	10,824 (7.8%)	
Malay (%)	14,582 (9.9%)	535 (7.4%)	14,047 (10.1%)	
Others (%)	5804 (5.9%)	167 (2.3%)	5637 (4.0%)	
Social Determinants of Health				
Resided in Public rental housing (%)	10,400 (7.1%)	1162 (16.0%)	9238 (6.6%)	< 0.001
Past 1-year Healthcare Utilization during first year of inclusion				
Public Primary Care Clinic visits, Mean (SD)	2.45 (4.16)	4.08 (7.14)	2.45 (4.15)	< 0.001
ED visits, Mean (SD)	0.15 (0.72)	2.87 (4.81)	0.14 (0.67)	< 0.001
Specialist Clinic visits, Mean (SD)	2.50 (5.65)	12.90 (16.50)	2.48 (5.59)	< 0.001
Hospital admissions, Mean (SD)	0.12 (0.54)	2.79 (3.75)	0.11 (0.50)	< 0.001
Medical Comorbidities^a^				
Diabetes without complications (%)	20,808 (14.1%)	2605 (35.4%)	18,203 (13.0%)	< 0.001
Hypertension (%)	43,057 (29.3%)	4802 (65.3%)	38,255 (27.4%)	< 0.001
Hyperlipidaemia (%)	42,437 (28.8%)	4090 (55.6%)	38,347 (17.9%)	< 0.001
Chronic Kidney Disease Stage 3–4 (%)	4614 (3.1%)	1255 (17.1%)	3359 (2.4%)	< 0.001
Asthma (%)	4958 (3.4%)	359 (4.9%)	4599 (3.3%)	< 0.001
Chronic Obstructive Pulmonary Disease (%)	3085 (2.1%)	681 (9.3%)	2404 (1.7%)	< 0.001
Chronic Obstructive Pulmonary Disease with cor pulmonale (%)	2574 (1.8%)	548 (7.5%)	2026 (1.5%)	< 0.001
Osteoarthritis (%)	16,787 (11.4%)	1186 (16.1%)	15,601 (11.2%)	< 0.001
Diabetes with complications (%)	2169 (1.5%)	434 (5.9%)	1735 (1.2%)	< 0.001
Cerebrovascular accident (%)	5173 (3.5%)	1355 (18.4%)	3818 (2.7%)	< 0.001
Chronic kidney disease stage V or End-stage renal failure (%)	1807 (1.2%)	827 (11.2%)	980 (0.7%)	< 0.001
Depression (%)	2810 (1.9%)	303 (4.1%)	2507 (1.8%)	< 0.001
Schizophrenia (%)	561 (0.4%)	108 (1.5%)	453 (0.3%)	< 0.001
Dementia (%)	513 (0.4%)	268 (3.6%)	245 (0.2%)	< 0.001
Collagen vascular disease (%)	517 (0.4%)	102 (1.4%)	415 (0.3%)	< 0.001
Parkinson disease (%)	481 (0.3%)	185 (2.5%)	296 (0.2%)	< 0.001
Epilepsy (%)	715 (0.5%)	138 (1.9%)	577 (0.4%)	< 0.001
Coronary heart disease (%)	9509 (6.5%)	2035 (27.7%)	7474 (5.3%)	< 0.001
Atrial fibrillation (%)	1286 (0.9%)	479 (6.5%)	807 (0.6%)	< 0.001
Heart failure (%)	2196 (1.5%)	910 (12.4%)	1286 (0.9%)	< 0.001
Peripheral vascular disease (%)	1124 (0.8%)	355 (4.8%)	769 (0.6%)	< 0.001
Hip fracture (%)	279 (0.2%)	113 (1.5%)	166 (0.1%)	< 0.001
Spine fracture (%)	452 (0.3%)	125 (1.7%)	327 (0.2%)	< 0.001
Chronic liver disease (%)	1074 (0.7%)	225 (3.1%)	849 (0.6%)	< 0.001
Pressure ulcer (%)	243 (0.2%)	145 (2.0%)	98 (0.07%)	< 0.001
Malignancy (%)	4893 (3.3%)	1297 (17.6%)	3596 (2.6%)	< 0.001

*SD* standard deviation, *ED* emergency department, *ICD* international classification of diseases

^#^Continuous variables were analyzed using Student’s -test and categorical variables were analysed using chi-square test or Fisher’s exact test when appropriate

^a^Based on ICD codes in the preceding five years

Table 2 shows the results of multivariate cox regression analyses. After adjustment for covariates which included patients’ demographics, co-morbidities and past healthcare utilization, residence in public rental housing remained significantly associated with all-cause mortality [Hazard ratio (HR): 1.568, 95% confidence interval (CI): 1.469–1.673, *p* < 0.001]. Other demographic characteristics associated with increased mortality included age, male gender and Chinese ethnicity (*p* < 0.001). With the exception of comorbidities which included chronic obstructive pulmonary disease with cor pulmonale, depression, collagen vascular disease, atrial fibrillation, peripheral vascular disease and spine fracture, all other 20 co-morbidities examined in this study were associated with increased all-cause mortality (*p* < 0.05).

**Table 2:** Multivariable Cox regression analysis

Variable	Adjusted HR (95% CI)	*P*-value
Patient demographics		
Age	1.084 (1.082, 1.086)	< 0.001
Gender (Male)	1.581 (1.508, 1.658)	< 0.001
Ethnicity		
Others	Reference	
Chinese	1.114 (1.009, 1.230)	0.033
Indian	1.481 (1.353, 1.621)	< 0.001
Malay	1.041 (0.891, 1.217)	0.613
Social Determinants of Health		
Residing in Public Rental Housing	1.568 (1.469, 1.673)	< 0.001
Past One Year of Healthcare Utilization		
ED visits	0.983 (0.964, 1.001)	0.071
Specialist Clinic visits	1.015 (1.013, 1.017)	< 0.001
Hospital admissions	1.085 (1.060, 1.110)	< 0.001
Medical Comorbidities^a^		
Diabetes without complications	1.249 (1.177, 1.326)	< 0.001
Hypertension	1.083 (1.011, 1.160)	0.001
Hyperlipidaemia	0.653 (0.612, 0.698)	0.034
Chronic Kidney Disease Stage 3–4	1.308 (1.181, 1.449)	< 0.001
Asthma	0.869 (0.769, 0.983)	0.025
Chronic Obstructive Pulmonary Disease	1.391 (1.164, 1.663)	< 0.001
Chronic Obstructive Pulmonary Disease with cor pulmonale	1.032 (0.850, 1.254)	0.749
Osteoarthritis	0.675 (0.632, 0.720)	< 0.001
Diabetes with complications	1.238 (1.113, 1.377)	< 0.001
Cerebrovascular accident	1.605 (1.502, 1.716)	< 0.001
Chronic kidney disease stage V or End-stage renal failure	1.546 (1.365, 1.752)	< 0.001
Depression	1.037 (0.914, 1.175)	0.575
Schizophrenia	1.835 (1.501, 2.244)	< 0.001
Dementia	1.365 (1.191, 1.565)	< 0.001
Collagen vascular disease	1.191 (0.968, 1.464)	0.098
Parkinson disease	1.589 (1.365, 1.850)	< 0.001
Epilepsy	1.792 (1.500, 2.140)	< 0.001
Coronary heart disease	1.244 (1.168, 1.326)	< 0.001
Atrial fibrillation	1.103 (0.993, 1.226)	0.066
Heart failure	1.721 (1.580, 1.874)	< 0.001
Peripheral vascular disease	1.299 (1.155, 1.462)	0.809
Hip fracture	1.434 (1.185, 1.736)	< 0.001
Spine fracture	1.031 (0.854, 1.245)	0.753
Chronic liver disease	1.796 (1.564, 2.063)	< 0.001
Pressure ulcer	1.390 (1.155, 1.673)	0.001
Malignancy	2.967 (2.786, 3.160)	< 0.001

*HR* Hazards ratio, *ED* emergency department, *ICD* international classification of diseases

^a^Based on ICD codes in the preceding five years

**Fig. 2:**
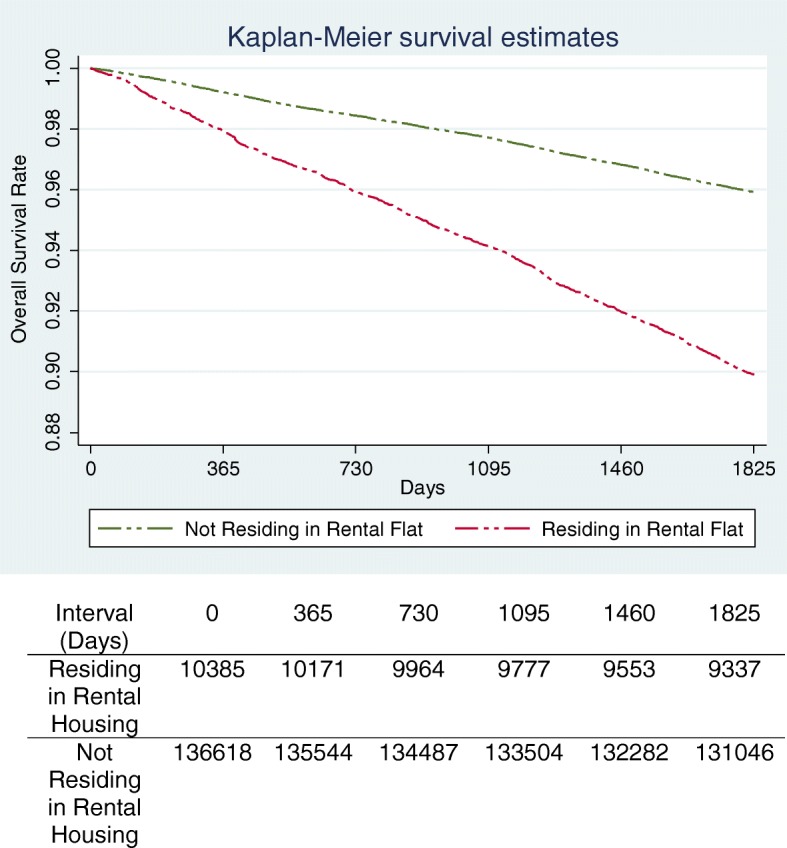
Kaplan-Meier curve for survival probability stratified by residence in public rental housing

Figure 2 shows the Kaplan Meier curve for all-cause mortality stratified by residence in public rental housing. The 5-year mortality of patients living in public rental housing was significantly higher (*p* < 0.001).

## Discussion

We found that residing in public rental housing was associated with increased all-cause mortality among patients, after adjustment for demographic and clinical characteristics. This concurred with findings from other studies which showed a positive correlation between low SES and adverse health outcomes [26, 27].

Underprivileged housing condition is tied closely to poorer health as inadequate household conditions due to overcrowding, sanitation, and poor indoor air quality often contribute to communicable diseases and exacerbations of chronic illnesses [6]. It is also a marker of low SES and social instability which compromises residents’ access to health care [6]. In Singapore, potential causes for our finding may be related to public rental housing residents’ lower health literacy, difficult financial conditions and health beliefs. Chan et al. summarized the health status, health seeking behaviour and healthcare utilisation of low socioeconomic status populations residing in public rental housing in Singapore [28]. A study by Wee et al. showed that public rental housing residents were more likely to seek medical attention when there is manifestation of bothersome symptoms such as chronic pain [29]. Another study found that the costs of screening and treatment were the chief barriers deterring public rental housing residents’ participation in health screening programmes [30]. Collectively, these may prevent early detection and treatment of chronic diseases and malignancies which increase their risk of mortality. In addition, studies have demonstrated a higher usage of alternative medicine as well as distrust in doctor-patient relationship among public rental housing residents, which may prevent them from seeking timely medical attention [16, 31]. Suboptimal housing conditions such as sanitation, poor indoor air quality and overcrowding in public rental housing may also contribute to poor health. For example, household air pollution has negative impacts on patients with chronic respiratory diseases such as asthma and chronic obstructive pulmonary disease [15]. However, similar comparative studies are not available in Singapore. Air quality and pollution is of a lesser concern as public rental housing in Singapore is formulated by housing policy to be integrated with more affluent housing communities and prevent the formation of ghettos.

The relationship of socioeconomic inequality and mortality is a complex and involves the interplay of material, behavioural and psychosocial factors which may vary over time [32, 33]. Residence in public rental housing has been suggested to affect the health of residents both positively and negatively. Postulated reasons for its positive effects on health are due to income, quality, gateway and network effects [9]. Income effect refers to the freeing up of income for procurement of health services, while quality effect refers to the tight regulation of public housing quality which minimizes residents’ exposure to lead and pest infestation [9]. Gateway effect refers to locating subsidised housing in close proximity to social service organisation and network effect refers to sharing of information within public rental residents as well as social support [9]. The positive effects of public rental housing could not be evaluated in this study and may be considered in future studies.

Unsurprisingly, we found that patients who died during study period had higher rates of co-morbidities such as hypertension and hyperlipidaemia. High disease burden is a well-recognized predictor of mortality among patients with different disease states [34]. It is noteworthy that the prevalence of chronic obstructive disease (COPD) (9.1%) among patients who died was significantly higher than the national average of 3.5% [35]. After adjustment for covariates, COPD was also associated with increased risk of mortality. A study by Brugge et al. showed a positive correlation between increased respiratory symptoms and residence in public housing [36]. Some of the contributing factors suggested for poorer respiratory health status included environmental and social factors such as mold, poor hygiene and smoking in the household. Given the well-established association of smoking with malignancies and other metabolic diseases [37], future studies should explore if smoking is a prevalent problem among public rental housing residents to evaluate the need for implementation of targeted smoking cessation programs. In Singapore, cleanliness of common areas within housing estates is maintained by government town councils. However, each resident is responsible for the internal cleanliness of their flats. Studies may wish to consider exploring the hygiene level within the living quarters of public rental housing residents and its potential impact on residents’ health outcomes.

Interestingly, depression was not associated with increased mortality among patients. This contrasted findings from by Reynolds et al. who found that depressive symptoms was associated with shortened life expectancy [38]. A potential reason for the differing findings could be due to the age differences between the study populations. Patients included in this study were comparatively younger (50.2 ± 17.2 years old) than patients included in the Florida study which involved geriatric patients aged ≥70 years old [38].

Overall, while public rental housing was found to be an independent risk factor for mortality, interpretation of results should also take into account Singapore’s unique housing and healthcare policies. More than 80% of the home ownership in Singapore is accounted by public housing sold under long-term lease. Compared to Hong Kong, another urbanized Asian city, where 31% of households resides in public rental housing [39], the proportion of households residing in public rental housing in Singapore is lower (6%). Universal healthcare coverage is also provided to all Singapore citizens through a mixed financing system, which is achieved through compulsory medical savings for individuals, utilization of market-based mechanisms and technology to improve healthcare outcomes [40].

Our study also had several limitations. Firstly, variables that could be analysed in the study included only routinely collected data from electronic databases within SHRS. Consequently, we were unable to evaluate the differential causes of mortality and other socio-demographic variables such as history of smoking and income level in the study. Factors that have been linked with poorer health outcomes among patients with lower SES such as dietary quality, level of physical activity, health literacy and education level could not be assessed [41–44]. These factors should be evaluated in future studies. In addition, we were also unable to establish a causal association between public rental housing and mortality due to the retrospective nature of the study.

## Conclusion

We found that public rental housing was an independent risk factor for all-cause mortality. More studies should be conducted to understand the health-seeking behaviours, healthcare needs and social circumstances of public rental housing residents. This will aid policy makers in formulating better policies to improve the health-related outcomes for this population.

## Abbreviations

CIRB: Centralized Institutional review board
ICD: International classification of diseases
SES: Socioeconomic status
SRHS: Singhealth regional health system
